# Conformational and Functional Effects Induced by D- and L-Amino Acid Epimerization on a Single Gene Encoded Peptide from the Skin Secretion of *Hypsiboas punctatus*

**DOI:** 10.1371/journal.pone.0059255

**Published:** 2013-04-02

**Authors:** Mariana T. Q. de Magalhães, Eder A. Barbosa, Maura V. Prates, Rodrigo M. Verly, Victor Hugo O. Munhoz, Ivan E. de Araújo, Carlos Bloch

**Affiliations:** 1 Laboratório de Espectrometria de Massa, Embrapa Recursos Genéticos e Biotecnologia, Brasília-Distrito Federal, Brasil; 2 Departamento de Biologia Celular, Pós-Graduação em Biologia Molecular, Universidade de Brasília, Brasília, Distrito Federal, Brasil; 3 Instituto de Química, Universidade Federal de Minas Gerais, Belo Horizonte, MG, Brasil; 4 The John B. Pierce Laboratory, New Haven, Connecticut, United States of America; 5 Department of Psychiatry, Yale University School of Medicine, New Haven, Connecticut, United States of America; 6 Departamento de Química Universidade Federal dos Vales do Jequitinhonha e Mucuri, Diamantina, Minas Gerais, Brazil; Northwestern University, United States of America

## Abstract

Skin secretion of *Hypsiboas punctatus* is the source of a complex mixture of bioactive compounds where peptides and small proteins prevail, similarly to many other amphibians. Among dozens of molecules isolated from *H. punctatus* in a proteomic based approach, we report here the structural and functional studies of a novel peptide named Phenylseptin (FFFDTLKNLAGKVIGALT-NH_2_) that was purified as two naturally occurring D- and L-Phes configurations. The amino acid epimerization and C-terminal amidation for both molecules were confirmed by a combination of techniques including reverse-phase UFLC, ion mobility mass spectrometry, high resolution MS/MS experiments, Edman degradation, cDNA sequencing and solid-phase peptide synthesis. RMSD analysis of the twenty lowest-energy ^1^H NMR structures of each peptide revealed a major 90° difference between the two backbones at the first four N-terminal residues and substantial orientation changes of their respective side chains. These structural divergences were considered to be the primary cause of the *in vitro* quantitative differences in antimicrobial activities between the two molecules. Finally, both molecules elicited equally aversive reactions in mice when delivered orally, an effect that depended entirely on peripheral gustatory pathways.

## Introduction

According to Stahl [1], multifunctional drugs are agents with more than one therapeutic mechanism and it has been observed that almost all existing drugs have more than one known pharmacological target when higher doses exceeding the remedial recommendations are administrated. Fine-tuned with Paracelsuś legendary quote, the multifunctional concept of a drug depends upon a given concentration to reveal its multiple pharmacological activities. Among many others, a classical example of this principle is Aspirin [2], the well-known drug used for more than a century as analgesic, antipyretic and anti-inflammatory, in the end 1960`s was also confirmed experimentally to be an effective inhibitor of platelet aggregation [3], therefore an important agent for ischemic stroke prevention [4]. Pondering these facts and more recent ones [5], it is reasonable to ask: why the multifunctional drug principle should be confined only to small molecules, natural products and/or their synthetic analogs? Would peptides and proteins also show similar properties under comparable experimental conditions? How many different structures, functional sites and therefore biological activities a single polypeptide chain would display after an extensive investigation?

A noteworthy indication towards a more comprehensive answer to these questions was given by Brandenburg and co-authors [6] in their recent review on antimicrobial peptides, when the multifunctional drug attributes of this class of molecules and a possible range of different applications were underlined.

The other end of the same spectrum show us that in principle, from the organism perspective, any exogenous substance alone may represent a potential hazardous to its integrity and/or survival. Living beings are equipped with numerous barriers, defense strategies and metabolic mechanisms to deal with physical, chemical and biological threats. In all that, small and macromolecules may be found acting in both ways, as belligerent and defense agents depending on the situation. It is interesting to note that animals may engender complex multimodal signals combined with volatile and toxic substances that play critical roles in defense strategies as well as in social interactions of a number of animal species [7]–[10]. In plants, it was demonstrated that homoterpene could also be released from damaged aerial tissues induced by herbivore attacks revealing a signaling mechanism similar to those in animals [11], [12]. In other words, many molecules may have multiple functions and activities depending on the metabolic target and relative concentration.

As for amphibians, it is widely known that they protect themselves against various biotic hazards using an intricate collection of strategies including noxious secretions, aposematism, deimatic behavior and evasive tactics [13]. The granular glands in anurans skin store extraordinary quantities of toxic compounds [14]–[17], which are also the most evident molecular ingredients of some aversive chemosensory signals released against potential predators to reduce the chances of future attacks [18], [19], as exemplified by the fact that some amphibian eggs and larvae are perceived as repulsive by some fish and birds [20]–[22]. Amongst the great variety of substances secreted by these glands, several of them are antimicrobial peptides [15], [23]–[28] typically characterized by basic, linear and amphiphilic primary structures of 30–35 residues in a single-chain [27], [29]–[31]. Although their protecting roles as microbicidal agentes have been thoroughly investigated during previous decades, little is known about other biological activities that antimicrobial peptides may display [32], [33]. In the present work, we report the ^1^H NMR structures and functional studies of a novel antimicrobial peptide that also displays aversive gustatory properties in mice models. The peptide naturally occurring in two enantiomeric configurations was isolated from *Hypsiboas punctatus* skin secretion and sequenced by a combination of ion mobility mass spectrometry, Edman degradation and cDNA methodologies.

## Materials and Methods

### 1. Amphibian Skin Secretion

Adult specimens of *H. punctatus* were collected in Palmas, Tocantins, Brazil. All procedures in this study were approved and performed are under the license IBAMA 0637/91 AC. The frog skin glands were subjected to mild electrical stimulation (6 V during 1 min) and skin secretion was collected in ice-cold Milli-Q H_2_O, filtered, and freeze-dried and stored at −80°C, as described by Prates, *et al* 2004 [34], [35].

### 2. Peptide Purification

The crude extract was dissolved in aqueous 0.1% trifluoroacetic acid (TFA), then fractionated by RP-HPLC (Class VP Shimadzu Co., Japan) using a Vydac® 218TP510 semi-preparative C_18_ column (Grace Vydac® TP, USA). The fractions were eluted in a 120 minutes linear gradient of Solvent A (0.1% TFA in Milli-Q H_2_O) and Solvent B (0.1% TFA in acetonitrile) using a 2.5 mL_ ˙_min^−1^ flow rate. Chromatographic fractions containing peptides of interest were submitted to further purification steps on analytical columns Vydac® 218TP54 C_18_ (Grace Vydac® TP, USA), Source™ 5 RPC ST (GE Healthcare, WI), and Shim-Pack-XR-ODS (30.0 mm×2.0 mm) C_18_ column on an Ultra-Fast-Liquid-Chromatographer (UFLC Prominence System Shimadzu Co., Japan). All the experiments were monitored at 216 and 280 nm (or 254 nm when phenylalanine residues were present), collected manually, frozen, lyophilized, and stored at −20°C.

### 3. Mass Spectrometry Analysis and Peptide Sequencing

The accurate molecular mass values of the peptides were determined by ESI-micrOTOF-Q II (Bruker Daltonics, Germany). The purity of each chromatographic fraction was evaluated by MALDI-TOF/MS (UltraFlex III, Bruker Daltonics, Germany) using close external calibration under reflector mode. Approximately 20 nM of lyophilized peptide was dissolved in Milli-Q H_2_O, mixed to a saturated solution of α-cyano-4-hydroxycinnaminic acid, spotted on a MALDI sample plate, and dried at room temperature. Peptide fragmentation was obtained by MALDI-TOF MS/MS experiments and the resulting data were analyzed manually using both Pepseq [36] and Flex Analysis 3.0 (Bruker Daltonics) softwares. The synthetic peptides were analyzed with a high-capacity ion trap LC/MSn system HCT Ultra ETD (Bruker Daltonics, Germany) equipped with a standard ESI ion source, with full scan data in UltraScan mode between m/z 100–3000, in positive mode, and with a fixed accumulation time of 30 ms. Additionally, amino acid sequencing was performed by the automated Edman degradation method on a PPSQ-23 protein peptide sequencer (Shimadzu Co., Japan).

### 4. Structural Studies on Ion Mobility Mass Spectrometry

Experiments were performed on a both MALDI Synapt G1 and ESI G2 HDMS instrument (Quadrupole Ion Mobility High-Definition mass spectrometry – Waters Co. MA, USA) equipped with nano-electrospray ionization source. All spectra were acquired by direct infusion of 1 µL·min^−1^ at a range of 300 up to 2000* m/z*. Cross section calculations of Phenylseptin were conducted as described [37].

### 5. Nucler Magnetic Resonance (NMR)

The sample was prepared dissolving the peptide at 2 mM in a 60% TFE/D_2_O (v/v) solution. The NMR experiments were conducted at 20°C on a Bruker Avance III spectrometer (Bruker DRX-800) operating at 800 MHz for the 1H frequency. The Total Correlation Spectroscopy (TOCSY) spectra were acquired using the MLEV-17 pulse sequence. The spectral width was determined as 6,900 Hz, and the 512 t_1_ increments were collected with eight transients of 4,096 points. NOESY spectra were acquired using mixing times of 80, 100, 120, 140 and 160 ms. For this experiment, the spectral width was 6,900 Hz and the 512 t_1_ increments were collected with 16 transients of 4,096 points for each F_1_. The ^1^H–^13^C HSQC spectra were acquired with F_1_ and F_2_ spectral widths of 27,160 and 8,993 Hz respectively. The 400 t_1_ increments were collected with 56 transients of 1,024 points. The acquisitions were carried out in an edited mode allowing CH and CH_3_ correlations could show a positive phase and CH_2_ correlations, a negative phase. The ^1^H–^15^N HSQC spectra were acquired with F_1_ and F_2_ spectral widths of 27,160 and 8,993 Hz, respectively. Eighty t_1_ increments were collected (with 400 transients of 1,024 points), for each free induction decay. NMR spectra were analyzed using NMRVIEW, version 5.0.3. NOE intensities obtained at 120 ms mixing times were semi-quantitative converted into distance restraints using the calibration described by [38]. The highest intensity peaks corresponded to restraints with upper limit of 2.8 Å, the medium intensity with 3.4 Å, and the weakest peaks, with 5.0 Å. The backbone atoms chemical shift values were used to generate the dihedral angle restraints, using the TALOS+ software [39]. Structures were calculated (200) using these restraints on the Xplor-NIH v.2.17 software [40] by simulated annealing protocol. The structure calculation started with an extended model, with 14000 steps at high temperature, 6000 steps during cooling and a time step of 0.005 ps. The generated structures were then submitted to a refinement procedure, using again a simulated annealing calculation, using the Internal Variable Module [41] available in Xplor-NIH, with the Hydrogen-Bonding Database, Ramachandran Torsion-Angle Database and the Carbon Chemical Shift potentials. Among the refined structures, the 20 lowest-energy ones were selected to represent the model ensemble and then validated on Procheck on-line software [42].

### 6. Gene Cloning, cDNA Sequencing and Analysis

Total RNA from *H. punctatus* was extracted using the Trizol reagent according to a previously described protocol [23]. Reverse transcription of total RNA (1 µg) was performed with d(T)-anchor primer and the Superscript Reverse Transcriptase kit™ (Invitrogen), according to the manufacturer’s instructions. PCR reactions were performed using degenerate primers designed based on conserved signal peptide [24]. The amplified cDNA fragments were cloned into pGEM-T easy® (Promega, Maldison, WI, USA) and sequenced in both strands using an ABI Prism 3700 DNA Analyzer system with BigDye™ terminator and POP-5™ Polymer (Applied Biosystem Perkin Elmer). Comparisons of the cloned cDNA sequence with other GenBank™ database sequences were performed using the BLASTp software [43] from the NCBI databank (http://www.ncbi.nlm.nih.gov). The Conserved Domain Database search (CDD-Search) from the NCBI site was used to compare motif identity and similarity with known conserved domains [44]. Sequence alignments were obtained by CLUSTAL W software [45] and were edited using BIOEDIT [46]. The calculated molecular mass of deduced proteins and sequenced peptides were determined by the Protein Machine software available at the Expasy website (http://us.expasy.org/tools/).

### 7. Solid Phase Peptide Chemical Synthesis

The peptides L-Phes, D-Phes and one synthetic short-analog L-Phes.1 were manually synthesized by solid phase using the Fmoc/t-butyl strategy. An Fmoc-PAL-PEG-polystyrene resin was used for synthesis of the amidated C-terminal segment. Cleavage and final deprotection were conducted with a TFA:thioanisole:ethanedithiol:triisopropylsilane (91.5∶5∶2.5∶1, v:v:v:v) solution for 1 hour at room temperature. Peptide purification was performed through RP-HPLC with a Vydac 218TP1022 preparative column and purity was assessed by MALDI-TOF/MS.

### 8. Antimicrobial Assays

The antimicrobial activity of the peptides L-Phes and D-Phes were investigated using the bacterial strains *Pseudomonas aeruginosa* ATCC 27853, *Staphylococcus aureus* ATCC 43300, and *Escherichia coli* ATCC 25992. In addition, we investigated the antimicrobial activity of the peptides towards a phytopathogenic bacterium *Xanthomonas. axonopodis pv glycines ISBF 327* obtained from Embrapàs microorganism collection. All microorganisms were grown in stationary culture at 37°C and after that were transferred to Mueller-Hinton liquid medium, according to the National Committee for Clinical and Laboratory Standards (NCCLS) to perform the bioassays (NCCLS Institute protocols). The peptide was dissolved up to 8-fold in Mueller-Hinton liquid broth. The highest peptide concentration used in the assay was 131 µM in an initial inoculum of 2.5.10^5^ cfu·mL^−1^ (colony-forming units/mL). The final volume was 100 µL per well, 50 µL of the peptide and 50 µL of the inoculum. The experiment was carried out in stationary culture at 37°C, and the spectrophotometer readings were performed 12 h after incubation. The minimal inhibitory concentration (MIC) was determined based on three independent measurements, using the optical density parameter (A_595_ nm). Conventional antibiotics (ampicillin and chloramphenicol) had their minimum inhibitory concentrations determined against the three experimental bacterial strains.

### 9. Behavioral Gustatory Assays

Male, adult (8–10 weeks old) mice on a C57BL/6 background were used for behavioral assays. Animals used in this study included littermates generated from mice heterozygous for a partial deletion of the *Trpm5* gene, as previously described [47], originally bred from mice generously donated by C. S. Zuker (UCSD, San Diego, CA). Genotype was confirmed by PCR amplification using specific primers for *Trpm5* gene (forward 5′-ATTCTAGAGCCCACCCGCCCCATC-3′ and reverse 5′-TTCACCTGCCCAGCCCTCATCTAC-3′) and for PGK-neor cassette that replaced exons 15 to 19 encoding the first five transmembrane domains of *Trpm5* in mutant animals (forward 5′-TTGCACGCAGGTTCTCCGGC-3′ and reverse: 5′-TAGAAGGCGATGCGCTGCGA-3′). Heterozygous mice were identified by amplification of both the *Trpm5* gene and PGK-neor cassette; knockout mice were identified by the absence of *Trpm5* gene but amplification of PGK-neor cassette; and wild-type animals by absence of PGK-neor cassette but positive *Trpm5* gene amplification. Behavioral experiments were conducted in two mouse behavior chambers enclosed in a ventilated and sound-attenuating cubicle (Med Associates Inc., St. Albans, VT). Each chamber was equipped with two slots for sipper tubing placement in symmetrical locations of one of the walls. All slots were equipped with licking detection devices (contact lickometers, Med Associates Inc., St. Albans, VT, USA) performing with a 10 ms resolution. All experiments were conducted in accordance with the J.B. Pierce Laboratory and Yale University regulations on usage of animals in research. All procedures performed in this study were approved by the Animal Care and Use Committee of The J. B. Pierce Laboratory.

#### 9.1 Controls and stimulus concentrations

As a positive control, well known plant bitter alkaloids were used: caffeine, theophylline, and theobromine. Stimulus concentrations were based on previous studies, which reported, in rats, that the threshold for integrated cranial nerve responses is 10 mM for the glossopharyngeal nerve and approximately 10–30 mM for the chorda tympani [48]. Accordingly, we used 10–20 mM (as well as the sub-threshold concentration of 5 mM) in our studies to evaluate the role of *Trpm5* in generating behavioral responses to caffeine. Finally, to allow for comparisons between behavioral responses across controls on a molar basis, we have used these same concentrations in theophylline and theobromine experiments. As a negative control we used free amino acid mixtures corresponding to the peptide primary structures. Stimulus concentrations were selected based on experiments conducted by Maehashi and Huang, 2009, where it was shown that a 10–20 mM threshold of free-amino acid is able to display bitter response in mice and rats. For the peptides L-Phes, D-Phes, and L-Phes.1 we considered the same concentrations as starting points.

#### 9.2. Short-term two-bottle preference tests

Once habituated to the behavioral chamber, each animal was presented with two bottles, contained either water or the stimulus solution prepared in distilled water. Animals were given free access to both bottles during the experiment (10 minutes). The number of licks for each sipper was recorded and used to calculate the preference ratios (see below). To reduce confounds associated with side-biases, mice were tested in each condition for four consecutive days with daily inversion of bottle positions. The average preference ratio across testing days was then calculated for each animal. Animals had been water-deprived for 16 hours previous to the beginning of each session.

#### 9.3. Data analysis

Results from data analyses were expressed as mean ± SD. Analyses of behavioral data were performed with the custom software Origin Professional (v8, OriginLab, Northampton, MA) using 2-way or 1-way ANOVAs followed by two-sample or independent one-sample t-tests. Bonferroni corrections for multiple comparisons were performed whenever appropriate.

#### 9.4. Lick response and preference measures

All two-bottle preference tests were analyzed by calculating the preference ratios as:
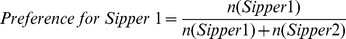
Where *n(.)* denotes the total number of licks counted on a given sipper during a session. These values were subjected to a 2-way ANOVA genotype × stimulus concentration model and tested against 0.5, which is the reference value meaning indifference with respect to water.

## Results

### 1. Phenylseptins on H. punctatus Skin Secretion

**Figure 1:**
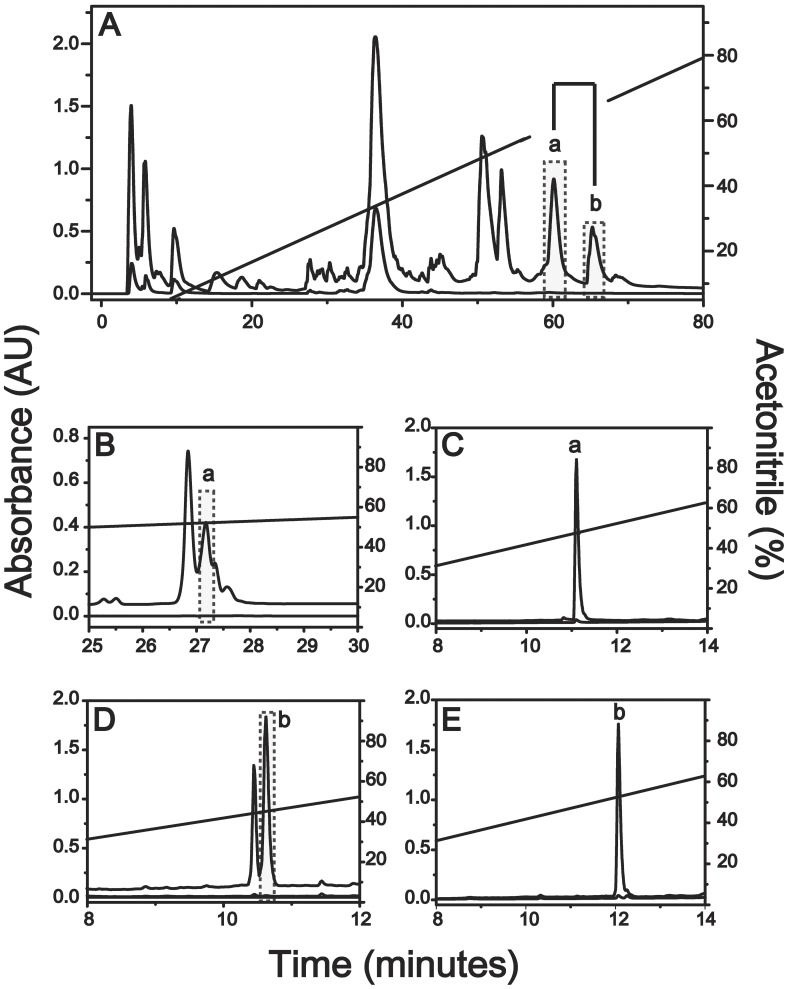
Phenylseptin peptides present on H. punctatus skin secretion. Chromatographic profile of H. punctatus crude extract and Phenylseptin isolation. (A) The crude extract was eluted with 0.1% TFA (Solvent A) and 95% acetonitrile containing 0.1% TFA (Solvent B), under a linear gradient of solvent B for 120 minutes in a semi-preparative C18 column. The identified Phenylseptin peptides ‘a’ and ‘b’ (B and D, respectively) were further purified by reverse-phase polystyrene/divinyl benzene chromatography using a 5 RPC ST 4.6 mm/150 mm column under optimized gradient of acetonitrile at a flow rate of 1.0 mL·min-1. After these two separation steps, final purification of the peptides ‘a’ and ‘b’ (C and E, respectively) was obtained using Ultra Fast Liquid Chromatography using a Shimpack-XR-ODS column under a linear gradient of acetonitrile at a flow rate of 0.4 mL·min-1. In all steps of purification the absorbance was measured at 216 nm, and when necessary at 254 or 280 nm.

**Figure 2:**
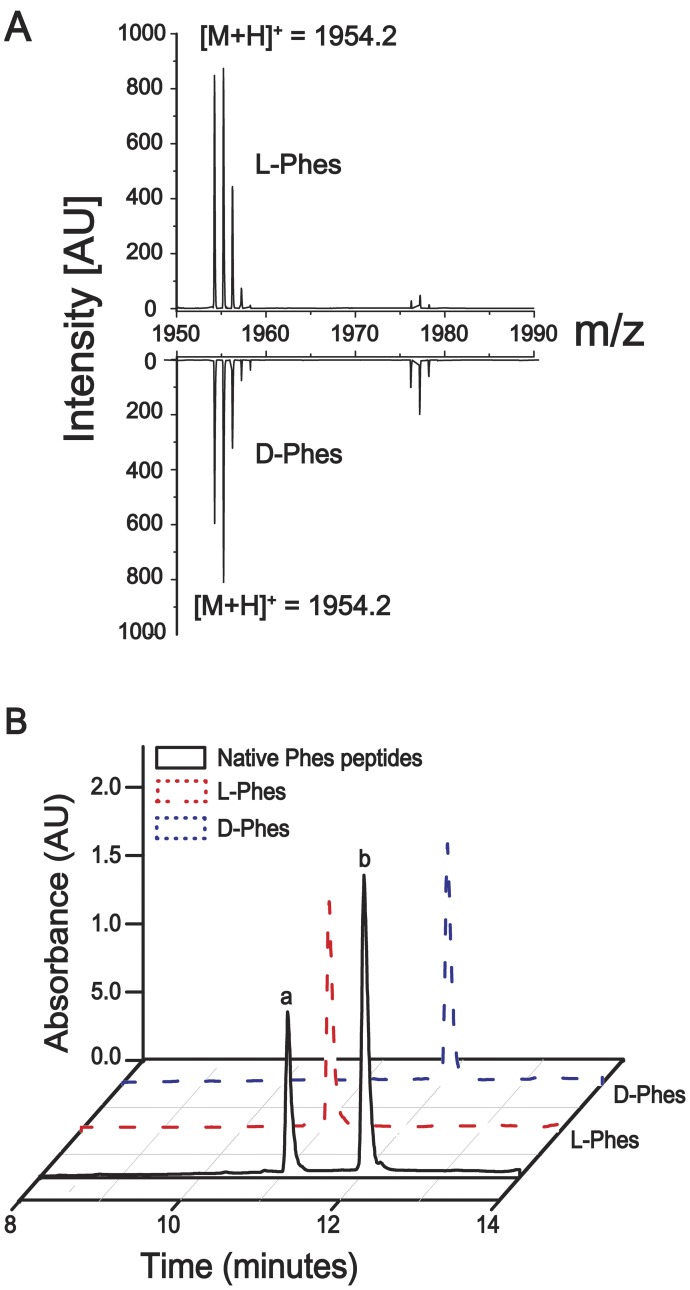
UFLC analysis and molecular mass determination of Phenylseptin mixture. (A) The accurate molecular masses and purity of Phes peptides were determined by MALDI-TOF/MS and the observed molecular mass was 1954.2 Da for both molecules. (B) Analytical chromatographic profile of natural (black line) and synthetic L-Phes (red dash line) and D-Phes (blue dash line). The two peptides were mixed in similar molar concentrations and load into an Ultra Fast Liquid Chromatography using a Shimpack-XR-ODS column under a linear gradient of acetonitrile at a flow rate of 0.4 mL·min-1. The two distinct fractions eluted around 11 and 12 minutes corresponded to L-Phes and D-Phes, respectively.

**Figure 3:**
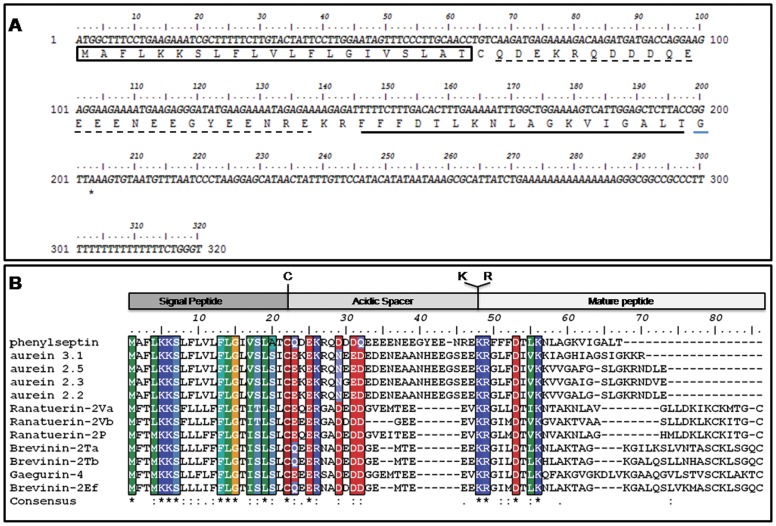
One single gene encoding Phes peptide. (A) Nucleotide sequences of clone encoding precursor of selected Phenylseptin peptides. The putative signal peptide (in box), acidic spacer (dash underline) and mature peptide (bold underline), C-terminal codon for Glycine (blue underline) and stop codon (asterisk) are indicated. The nucleotide sequences were deposited in the NCBI Nucleotide Sequence Database under HQ012497 annotated accession code. (B) Predicted amino acid sequence alignment of Phenylseptin with previously sequenced Hylidae peptides aurein, ranateurin, brevinin and gaegurin. Sequence alignments were done using CLUSTAL W software and were edited with the BIOEDIT software.

The crude skin secretion was fractionated by semi-preparative reverse-phase HPLC and mass analyzed by both MALDI TOF-TOF/MS and ESI Q-TOF/MS systems yielding about 20 major fractions (Figure 1A) from which, components “a” and “b” eluted at 61.0 and 64.3 minutes, respectively, showed identical molecular masses and MS/MS spectra. Each of the fractions was submitted to further purification steps on analytical scale to attain homogeneous peptide samples (Figure 1B–1E), confirmation of their distinct retention times, MALDI-TOF/MS monoisotopic molecular mass analyses (M+H^+^ = 1954.20 Da, Figure 2A) and primary structure elucidation from their corresponding MS/MS data (Figure S1). For both components (a and b), *de novo* sequencing based on the resulting sets of undistinguishable daughter ion series revealed 100% similarity between the amino acid sequences of the two components (see Table S1) bearing the following 18 residue polypeptide chain: FFFDTLKNLAGKVIGALT-NH_2_. Edman degradation of the intact components and their respective tryptic fragments excluded typical MS/MS data ambiguities regarding leucine, isoleucine, lysine and glutamine residues (Table S2). Moreover, the final sequence confirmation of both peptide and Thr18 C-terminal amidation was achieved by cDNA sequencing as shown in Figure 3 and ESI Q-TOF/MS exact mass of M+2H^+^ = 977.561 m/z (1.0 ppm) determinations (Figure S2).

### 2. One Single Gene Encoded Peptide

RT-PCR amplification produced various 320-bp fragments that revealed one single nucleotide sequence encoding a canonical antimicrobial pre-pro-peptide precursor (18) containing a deduced 18 residues peptide sequence (Figure 3A) identical to the previously obtained by mass spectrometry and Edman degradation. The pre-pro-peptide precursor also showed primary structure identity with antimicrobial sequences previously deposited in GenBank™ from three different Ranidae species, *Rana rugosa* (37% gaegurin), *Rana temporaria* (48% brevinin-2Ta and 46% 2Tb), *Rana esculenta* (50% brevinin-2Ef), and from one Hylidae specie, *Litorea aurea* (54% aurein 3.1; 56% aurein 2.5 and aurein 2.3; 57% aurein 2.2) (Figure 3B). The unusual triple phenylalanine residues at the N-terminal of the two putative antimicrobial peptides, their discrepancies in retention times by RP-HPLC and the confirmed complete sequence identity brought us to the hypothesis of a possible epimerization of at least one amino acid residue along the polypeptide chain, hence the name Phenylseptins for the two peptide analogs. The gene sequence was deposited under the genebank (gb|) accession number HQ012497.

### 3. D-phenylalanine is Present in Native D-Phes

Reverse-phase UFLC on a Shim-Pack-XR-ODS (2.0 mm *i.d*. × 30.0 mm) C_18_ column analyses determined that Phes ‘a’ was associated with a retention time of 11 minutes, and Phes ‘b’ with a retention time of 12 minutes (Figure 2B – black line). After enzymatic digestion with immobilized trypsin, UFLC analyses were performed to determine the retention times of all fragments from of these peptides (Figure S3 and Table S2). These analyses demonstrated that the fragments 1–7, corresponding to Phes ‘b’ N-terminal region (FFFDTLK; M+H^+^ = 917.55 Da) showed almost one minute difference in retention time when compared with the same fragment in Phes ‘a’. In order to investigate the presence of a D-phenylalanine residue at the second N-terminal position as suggested by previous related works [49]–[52], analogs containing [D-Phe2]-Phes and [L-Phe2]-Phes (henceforth D-Phes and L-Phes, respectively) were synthesized. After RP-UFLC native Phes ‘a’ co-eluted with L-Phes, while native Phes ‘b’ co-eluted with D-Phes (Figure 2B). In order to verify the presence of a D-phenylalanine residue at the N-terminal fragment, three possible analogues have been synthetized: [D-Phe1]-Phes, [D-Phe2]-Phes and [D-Phe3]-Phes. The three analogues (M+3H^+^977.7) were analyzed by RP-UFLC-MS and the individual retention time for each peptide was confirmed demonstrating that D-Phe2 analogue has similar retention time (over 12 min.) as the naturally occurring D-Phes peptide. (Figure S4).

### 4. Conformational Studies of Phenylseptin Analogs by Ion Mobility Mass Spectrometry (IM-MS)

**Figure 4:**
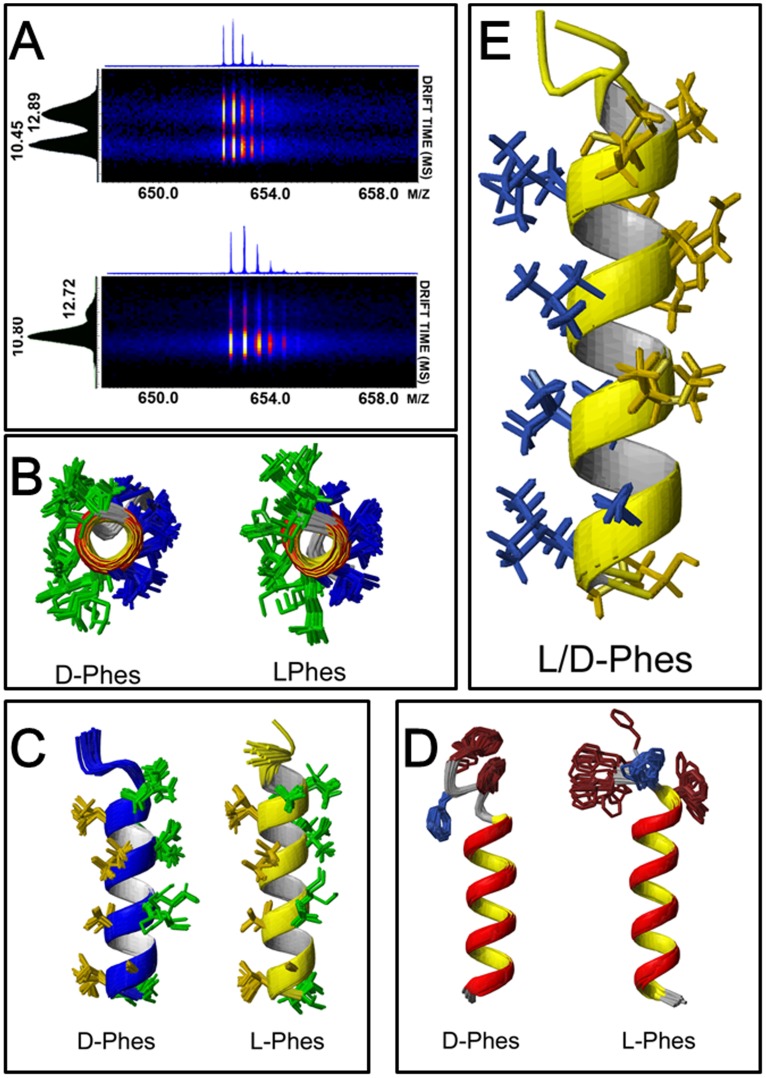
Structural studies on L- and D- Phenylseptin peptide isomers by Ion Mobility Mass Spectrometry (IM-MS) and Nuclear Magnetic Resonance (NMR). (A) L-Phes and D-Phes were individually analyzed showing that each one (M+3H^+^ = 652.04 m/z) can assume at least two major conformations with distinct amounts of each type. L-Phes conformations at 10.45 and 12.89 ms and D-Phes conformations at 10.80 and 12.72 ms. D-Phes has its major conformation at 10.80 ms. Experiments were performed on a Synapt HDMS instrument (Quadrupole Ion Mobility High-Definition mass spectrometry – Waters Co. MA, USA) equipped with nano-electrospray ionization. All spectra were acquired with a direct infusion of 1 µL·min-1 of in a range m/z 300 up to 2000. Precursor charge state: 3. Tolerance: 0.1 Da. (B) and (C) The 20 lowest-energy structures for both peptides. The hydrophobic residues are represented in gold yellow, the hydrophilic residues in green. (D) The lowest-energy Phenylseptins showing Phenylalanine enatiomerization, the aromatic phenylalanine are in dark red and (E) The alignment of lowest-energy L- and D-Phes structure in the presence of 60% TFE viewed along the helix axis and from the side.

In order to investigate possible conformational attributes of both Phenylseptins, these molecules were submitted to IM-MS analyses revealing at least two conformers (M+3H^+^ = 652.04 m/z) with comparable intensities for L-Phes and a major one for D-Phes with determined drift times of 10.45, 12.89 and 10.80 ms, respectively (Figure 4A). These results suggest higher degrees of freedom for the L-Phes structure in the gas phase in comparison to the D-Phes molecule.

### 5. NMR Structures of the Phenylseptins

Both NMR structures of L- and D-Phenylseptins at 60% TFE were produced using total sets of 361 and 373 NOEs, respectively (see Table 1 for details). NOEs correlations at the peptides three phenylalanine N-terminal region indicated significant structural differences between L- and D-Phes most likely due to Phe2 enantiomerization (Figure 4). Nevertheless strong NOEs correlations of the sequential dNN*_(i,i+1)_* and d*α*N*_(i,i+3)_* obtained for the rest of the molecule (residues 4–18) indicated classical α-helical conformations at that region for both peptides (Figure 4F–4G). The Root Mean Square Deviation (RMSD) analyses of all backbone atoms for individual structures were 0.53±0.21 for L-Phes and 0.38±0.18 for D-Phes, and no distance violations greater than 11 was observed. The RMSD values for segment 4–17 were similar between L-Phes and D-Phes (0.22±0.09 and 0.22±0.08, respectively), while L-Phes showed 0.40±0.22 for the^1^Phe-^3^Phe segment and D-Phes showed 0.08±0.06. In the calculated D-Phes structure it was observed that Phe1 and Phe3 aromatic rings have significant interactions whilst Phe2 share NOEs with the peptide side chain with Leu6 producing a more structured N-terminal than in L-Phes (Figure S5).

**Table 1:** Summary of the structural restraints and statistical analysis of the calculated structures of L/D-Phes in TFE/D2O 60:40 v/v.

Structural Statistics^a^	L-Phes	D-Phes
**Experimental restraints** b		
*Distance restrains*		
Intraresidue (i–j = 0)	185	184
Sequential (|i–j| = 1)	80	82
Medium range (2≤|i–j|≤4)	66	78
Long range (|i–j|≥5)	0	1
*Dihedral restrains*		
Torsion angle (phi/psi)	30	28
*Total number of restrains*	361	373
Restrains statistics		
NOE violations >0.5 Å	11	8
Dihedral violations >5°	3	1
**CNS energies (Kcal/mol)**		
E_total_	238.54±5.59	37.66±4.56
**RMSD from average for residues** **1–18 (Å)** c		
Backbone N, CA, C′	0.51±0.17	0.44±0.12
Heavy atoms	1.55±0.40	1.01±0.10
**RMSD from average for residues** **1–3 (Å)** c		
Backbone N, CA, C′	0.47±0.22	0.24±0.18
**RMSD from average for residues** **4–17 (Å)** c		
Backbone N, CA, C′	0.20±0.08	0.20±0.06
**Ramachandran plot** d		
Most favored regions (%)	85.7	99.6
Additional allowed regions (%)	0	0.4
Generously allowed regions (%)	8.9	0
Disallowed regions (%)	5.4	0

a The statistics was obtained from an ensemble of 20 lowest-energy vacuum-refined structures for both peptides (L/D-Phes).

b Restraint statistics reported for unique, unambiguous assigned NOEs.

c Coordinate precision is given as pair-wise Cartesian coordinate over the ensemble Root Mean Square Deviations from the average structure.

d Values obtained from the PROCHECK-NMR analysis over all residues including the first seven highly flexible residues.

### 6. Phenylseptins Display Different Antimicrobial Activities

*In vitro* antimicrobial tests revealed that both peptides exhibited distinct levels of activities against pathogenic *S. aureus* (ATCC 29313), *E. coli* (ATCC 25922) and *P. aeruginosa* (ATCC 27853). Overall, D-Phes was more effective than L-Phes and Magainin (classical positive control) [15] against these pathogenic bacteria but less active than DS01 [53], which was used as the most potent antimicrobial peptide (our groups positive control) (Table 2). When tested against the soybean phytopathogen, *Xanthomonas axonopodis pv glycines,* D-Phes was found to be 8-fold more effective than L-Phes (4.1 and 32.7 mM, respectively).

**Table 2:** Antimicrobial activity of L/D-Phes.

Microorganism	MIC (µM)^a^					
	L-Phes	D-Phes	Mg	DS01	Amp.	Chloram.
*S. aureus* ATCC 29313	65.5	32.7	ND	26,5	<11	ND
*E. coli* ATCC 25922	65.5	65.5	79	6,6	46	25
*P. aeruginosa* ATCC 27853	>130	130	157.8	NT	25	25
*X. axonopodis pv glycines* *ISBF 327* a	32.7	4.1	13	<1.42	ND	ND

a MIC -minimal peptide concentrations required for total inhibition of cell growth.

* ND - antimicrobial activity not detectable;

a Phytophatogenic bacteria; Amp and Chloram- ampicillin and chloramphenicol; Mg and DS01 - control peptides Magainin (Zasloff, *et al* and Brand, *et al*).

### 7. Bitterness Motif Hypothesis

**Figure 5:**
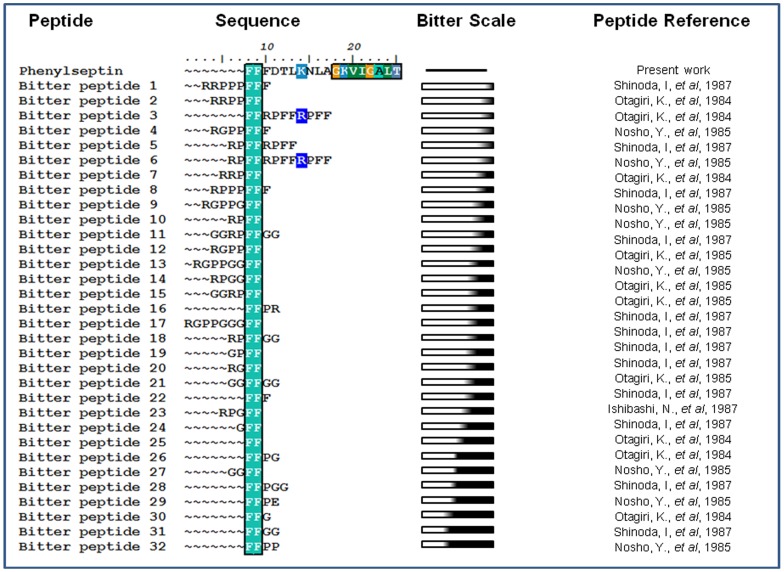
Phenylseptin Phe-Phe-Phe motif. Phenylseptin N-terminal sequence was compared to other peptides previously described on the literature as being bitter. Sequence alignments were edited with the BIOEDIT software. On the right side a bitterness scale based on Kim, *et al*., 2006 analysis.

Analyses of Phenylseptin sequences revealed that their Phe-rich N-terminals share sequence similarities with the bitter Phe-Phe-Phe motifs shown in Figure 5 [54]. Therefore, we hypothesized that the triple-phenylalanine N-terminal sequences found in the two peptides would also produce similar aversive behavioral responses to those produced upon stimulation of oral bitter taste sensors.

### 8. Gustatory Preference Tests Involving Prototypical Bitters in Trpm5 KO and WT Mice

**Figure 6:**
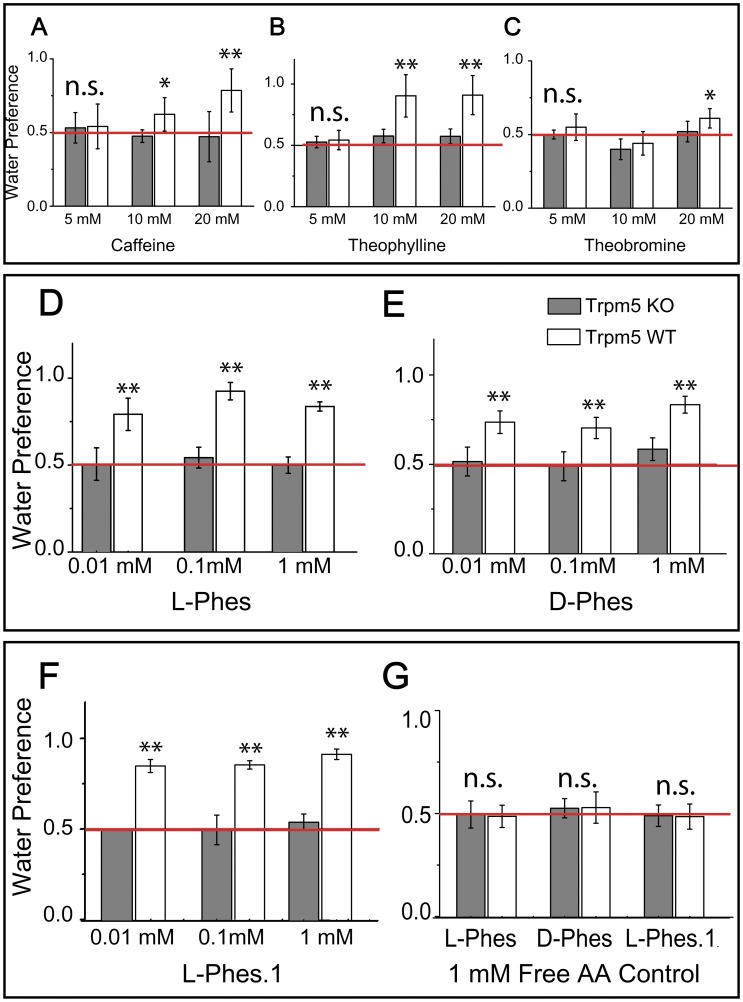
Trpm5 dependent bitter substances. Short-term (10 min) (A) caffeine vs. water, (B) theophylline and (C) theobromine two-bottle preference tests. Trpm5 knockout (“KO”) mice displayed indifference to all choices. (D) The bitterness of Phenylseptin peptides is dependent on Trpm5 regulation. Short-term (10 min) (E) L-Phes vs. water, (F) D-Phes vs. water and (3) L-Phes.1 vs. water two-bottle preference tests. Trpm5 knockout (“KO”) mice displayed indifference to all choices. Values displayed as preference ratios for water. (G) Amino acid organization on peptide structure is fundamental to gustatory perception. Short-term (10 min) L-Phes mixture vs. water and D-Phes mixture vs. water. Wild-type and Trpm5 knockout (“KO”) mice were equally indifferent to all choices. Values displayed as preference ratios for water. Values displayed as preference ratios for water. Post-hoc (Bonferroni-corrected) one sample t-test against indifference ratio of 0.5: *p<0.05; **p<0.04; all other comparisons p>0.05. Red line denotes level of indifference (preference ratio of 0.5).

The methylxanthines caffeine, theophylline and theobromine are prototypical bitter tastants and were used as positive controls for bitter detection in WT animals and bitter-insensitivity in KO animals at 5, 10 and 20 mM concentrations. For the caffeine experiments, we designed a mixed-model two-way stimulus concentration × genotype ANOVA × which revealed, as expected, a robust effect of genotype (p<0.0003) on preference ratios. Similar analyses performed for theophylline revealed equally robust effects of genotype on preference ratios (p<0.001). In addition, these ANOVAs revealed significant stimulus concentration × genotype interactions (p<0.02 and p<0.008; caffeine and theophylline, respectively). The interaction effects prompted repeated measures one-way ANOVA models for each genotype separately (Table S3), which revealed a significant main effect of stimulus concentration on preference ratios in WT (p<0.02 and p<0.01, caffeine and theophylline, respectively), but not in KO mice (p = 0.4 and p = 0.95, caffeine and theophylline, respectively). For theobromine this analysis revealed neither significant main effect of genotype (p = 0.17) nor interaction (p = 0.4) on preference rations, although it was the only stimuli to show a significant effect of stimulus concentration (p<0.02). One-way ANOVAs performed for each genotype separately confirmed a significant effect of theobromine concentrations in WT (p<0.04), but not in KO (p = 0.29). Post-hoc analyses of the individual preference ratios for each stimulus confirmed that, while WT mice displayed significantly higher preferences for water against 10–20 mM caffeine, 5-10-20 mM theophylline and 20 mM theobromine, KO mice were uniformly indifferent to all choices. Our findings so far show that, in 10 min-long two-bottle preference tests, *Trpm5 KO* mice are indifferent all concentrations of methylxanthines, while some concentrations (10–20 mM caffeine; 5–10–20 mM theophylline; 20 mM theobromine) are robustly avoided by WT mice. All control results are depicted in Figure 6A–6C and Table S3.

### 9. Gustatory Preference Tests Involving Phenylseptins in Trpm5 KO and WT Mice

After methodology validation, we tested both Phenylseptin peptides and one shorter analog. The analog L-Phes.1 was used as a control to investigate the importance of the full peptide sequence. We reasoned that the peptide N-terminal might be a crucial modulator of bitterness potency in these peptides. The peptides were tested at 0.01 mM, 0.1 mM and 1 mM in behavioral tests identical to those previously described. WT (N = 7) and KO (N = 7) mice were exposed to 10-min two-bottle preference tests where choice was given between a Phes solution and (distilled) water. Analysis of two-way genotype × peptide concentration ANOVA designed as above revealed a robust effect of genotype for L-Phes (F_1,12_ = 31.9, p<0.0002), D-Phes (F_1,12_ = 21.2, p<0.0006) and L-Phes.1 (F_1,12_ = 76.1, p<0.0005), on preference ratios. Conversely, no concentration effects were detected for L-Phes (F_1,12_ = 1.12, p = 0.31), D-Phes (F_1,12_ = 1.32, p = 0.27) or L-Phes.1 (F_1,12_ = 1.23, p = 0.30). Also, no significant peptide concentration × genotype interactions for L-Phes (F_1,12_ = 0.29, p = 0.75), D-Phes (F_1,12_ = 1.32, p = 0.27) or L-Phes.1 (F_1,12_ = 0.04, p = 0.84), on the preference ratios. Pairwise comparisons using post-hoc Tukey tests showed that even in low concentrations WT mice rejected the peptides, while KO mice remained indifferent. As observed for the control experiment with methylxanthines, post-hoc analyses on individual preference ratios of peptides confirmed that WT mice demonstrated high preference for water, while KO mice were indifferent to the choices given. Specifically, WT mice have shown significant higher aversion against all concentrations of L-Phes, D-Phes and L-Phes.1 (one-tailed t-tests, p<0.05; Figure 6D–6F). In summary, we attribute the aversive oral properties of the Phenylseptins tested entirely to their gustatory, possibly bitter-like, properties, given the strong impairments observed in mice lacking the taste ion channel TRPM5.

### 10. Peptide Primary Structure and Gustatory Perception

1 mM of each amino acid free present in L-Phes and D-Phes sequences were mixed and the resulting solutions were offered to the mice in two-bottle preference tests as above. The two-way ANOVA free-amino acid solution X genotype model showed no significant effect for L-Phes (p = 0.20), D-Phes (p = 0.19) and L-Phes.1 (p = 0.23), or interactions (L-Phes p = 0.37, D-Phes p = 0.41, and L-Phes.1 p = 0.35) on preference ratios for both preparations. One-way ANOVAs were performed separately for each genotype using the amino acid preparation as factor. There were no effects on preference ratios in either WT or KO mice. In fact, as expected for the tested concentration, both WT and KO mice were equally indifferent to 1 mM free amino acid solution (Figure 6G), indicating the importance of the primary structure for the activity.

## Discussion

The present work reports a novel peptide named Phenylseptin (Phes), purified from the skin secretion of *H. punctatus.* This peptide naturally occurs in two different primary structures constituted by L-Phe2 and D-Phe2 (Figure 2 and S3) and C-terminal amidation at Thr18. Both sequences display distinct *in vitro* antimicrobial activities but similar sensorial aversive properties on mice. It was observed that the epimerization of the second N-terminal phenylalanine residue induced a 90° conformational change at the N-terminal of the D-Phes structure (Figure 4) and that could be responsible for the significant differences on the antimicrobial activities obtained for two peptides (Table 1). However, the N-terminal structural differences demonstrated for both molecules appear to cause no detectable response regarding micès gustatory perception to bitterness. These findings are consistent with previous works concerning amino acid epimerization in frog peptides such as dermorphins and deltorphins, as well as predators aversive taste behaviors to various amphibian skin secretions [16], [51], [55]–[58].

The Phenylseptins reported in this study were found to adopt at least two major structural conformations and so far, according to our cDNA library, both forms should be considered a single gene product synthesized as a highly conserved pre-pro-protein that yields the two mature polypeptides (L−/D-Phes) after enzymatic processing and corresponding post-translational modifications. Electrospray and MALDI (data not shown) ion mobility mass spectrometry experiments demonstrated the presence of multiple peptide ion (M+H^+^) conformers in the gas phase (Figure 4A), which were confirmed by ^1^H NMR in solution (Figure 4D). The amount of structural data that was unveiled in this occasion for such a small polypeptide demonstrates how subtle, complex and non-trivial peptide studies can be, and how easily one might be driven to ignore relevant structural and functional features, particularly when high throughput approaches tend to be overemphasized. Furthermore, the uncommon triple phenylalanine N-terminal of the Phenylseptins seems to share similar bitterness attributes observed in peptides released during food hydrolysis or aging process in fermented products [59]. The aversive gustative responses registered in normal mice caused by aqueous solutions with different molar concentrations of Phenylseptins are comparable to those constituted by the classical bitter alkaloids caffeine, theophylline and theobromine, when offered to the same animals (Figure 6). Therefore, it is plausible to infer that similar aversive activity could also be part of a general warning strategy against natural predators of *H. punctatus*, especially if one may ponder what was uttered with remarkable precision in *De Anima* by Aristotle centuries ago: “…taste also must be a kind of touch, because it is a sensation of that which is tangible and nutritive… Hence excess in tangible qualities destroys not only the sense-organ, but also the animal itself. For touch is the one sense that the animal cannot do without… It (the animal) has taste on account of what is pleasant and painful, to the end that it may perceive what is pleasant in food and feels desire and be impelled to movement.” (Aristotle, III. 13) [60].

These results strongly suggest that Phenylseptins may be part of a multifunctional pool of peptides and proteins produced by the skin glands of *H. punctatus* with at least two experimentally tested defensive roles: against pathogenic microorganism and a potential predator warning agent.

## Supporting Information

Figure S1 **MS/MS spectra assignment for L-Phes and D-Phes fragmented peptides.** The observed molecular mass was 1954.2 Da. The peptides were fragmented by MALDI-TOF MS/MS experiments showing the same fragmentation profile. The resulting data were analyzed manually using both Pepseq (Waters Co.) and Flex Analysis 3.0 (Bruker Daltonics) programs. The primary sequence was confirmed by the automated Edman degradation method on a PPSQ-23 protein peptide sequencer (from Shimadzu Corp.). The loss of 0.98 Da on C-terminal threonine residue indicates the amidation.
(TIF)

Figure S2 **Theoretical and experimental accurate molecular mass values of Phenylseptins with internal calibration determined by direct infusion on an ESI MicrOTOF-Q II mass spectrometer operating with a standard ESI probe.** Mass accuracy of <1 ppm RMS error; M+2H^+^ = 977.651.
(TIF)

Figure S3 **Enzymatic digestion of natural L-Phes and D-Phes with immobilized trypsin and UFLC analyses.** (A) Analytical chromatographic profile of the digested peptides loaded onto an Ultra Fast Liquid Chromatography (UFLC-HPLC) using a Shimpack-XR-ODS column under a linear gradient of acetonitrile at a flow rate of 0.4 mL•min-1. L-Phes (red line) and D-Phes (blue line) generated 3 fragments each. Fragments 1/1′ and fragments 2/2′ were eluted at the same time, while fragments 3/3′ were eluted differently with a Δt = 0.9 min. (B) The molecular masses and purity of all fragments were determined by MALDI-TOF/MS (UltraFlex III, Bruker Daltonics, Germany) and the observed molecular mass was 524.1 Da for fragments 1/1′, 594.1 Da for fragments 2/2′ and 917.5 Da for fragments 3/3′.
(TIF)

Figure S4 **UFLC analyses of natural D-Phes and its analogues.** (A) Analytical chromatographic profile of the native (dash line) and synthetic (dark line) peptides loaded onto an Ultra Fast Liquid Chromatography (UFLC-HPLC) using a Shimpack-XR-ODS column under a linear gradient of acetonitrile at a flow rate of 0.4 m•min-1. L-Phes (red line) and D-Phes (blue line). (B) The molecular masses and purity of peptides were determined by ESI-HCT Ultra ETD (Iontrap, Bruker Daltonics, Germany) and the observed molecular mass was 977.7 Da for all analogues.
(TIF)

Figure S5 **NOESY spectra acquired using mixing times of 160 ms from D-Phes showing Phe2 and Leu6 interaction.** For this experiment, the acquisitions were carried out in 60% TFE/H_2_O (v/v).
(TIF)

Table S1 (DOCX)

Table S2 (DOCX)

Table S3 (TIF)
