# Supplementary figures and images for: Conformational and Functional Effects Induced by D- and L-Amino Acid Epimerization on a Single Gene Encoded Peptide from the Skin Secretion of Hypsiboas punctatus
Source: PLoS One. 2013 Apr 2;8(4):e59255. doi: 10.1371/journal.pone.0059255 (PMC3614549; doi:10.1371/journal.pone.0059255)

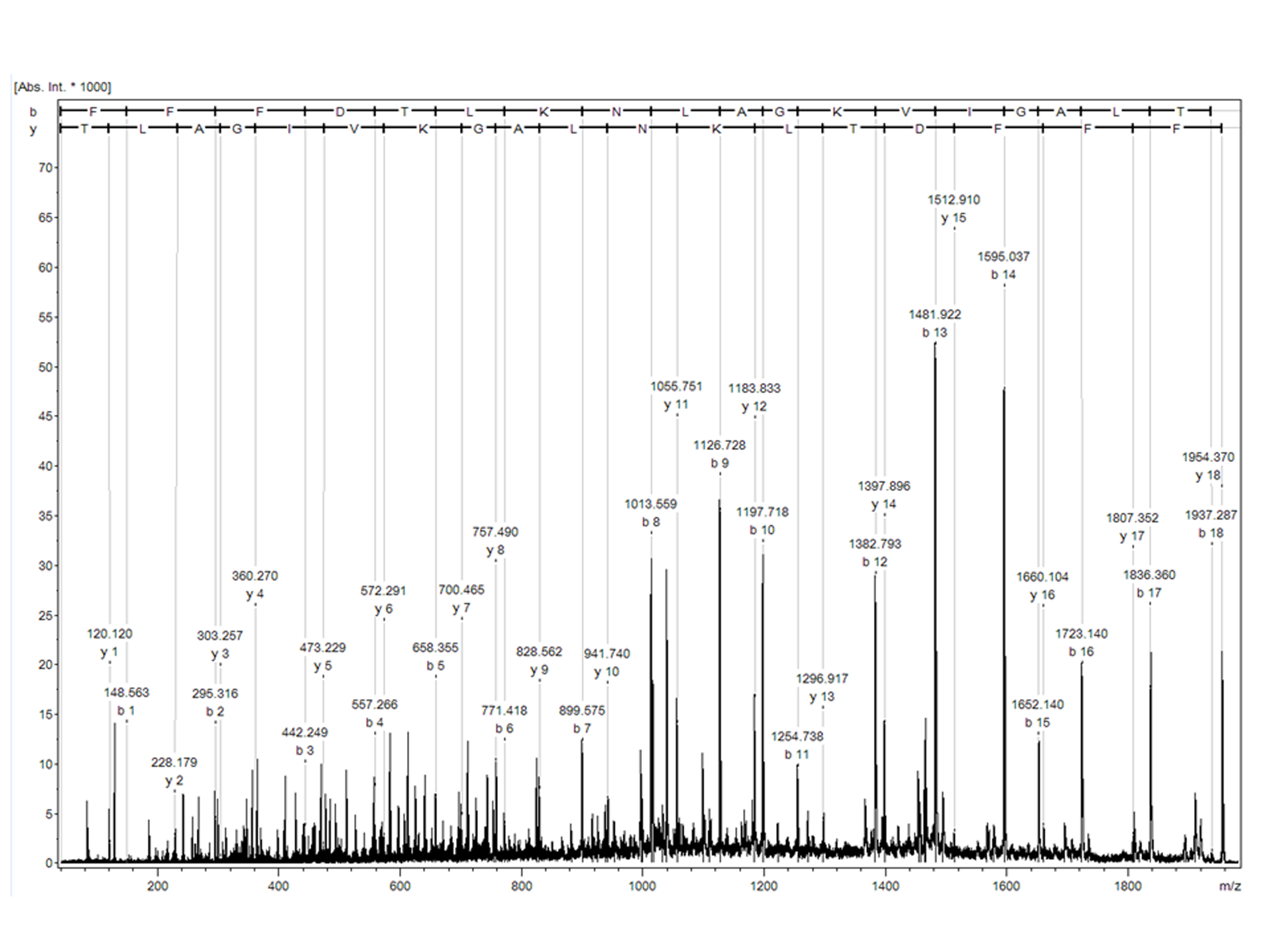

Supplement: Figure S1 — MS/MS spectra assignment for L-Phes and D-Phes fragmented peptides. The observed molecular mass was 1954.2 Da. The peptides were fragmented by MALDI-TOF MS/MS experiments showing the same fragmentation profile. The resulting data were analyzed manually using both Pepseq (Waters Co.) and Flex Analysis 3.0 (Bruker Daltonics) programs. The primary sequence was confirmed by the automated Edman degradation method on a PPSQ-23 protein peptide sequencer (from Shimadzu Corp.). The loss of 0.98 Da on C-terminal threonine residue indicates the amidation. (TIF) [file pone.0059255.s001.tif]

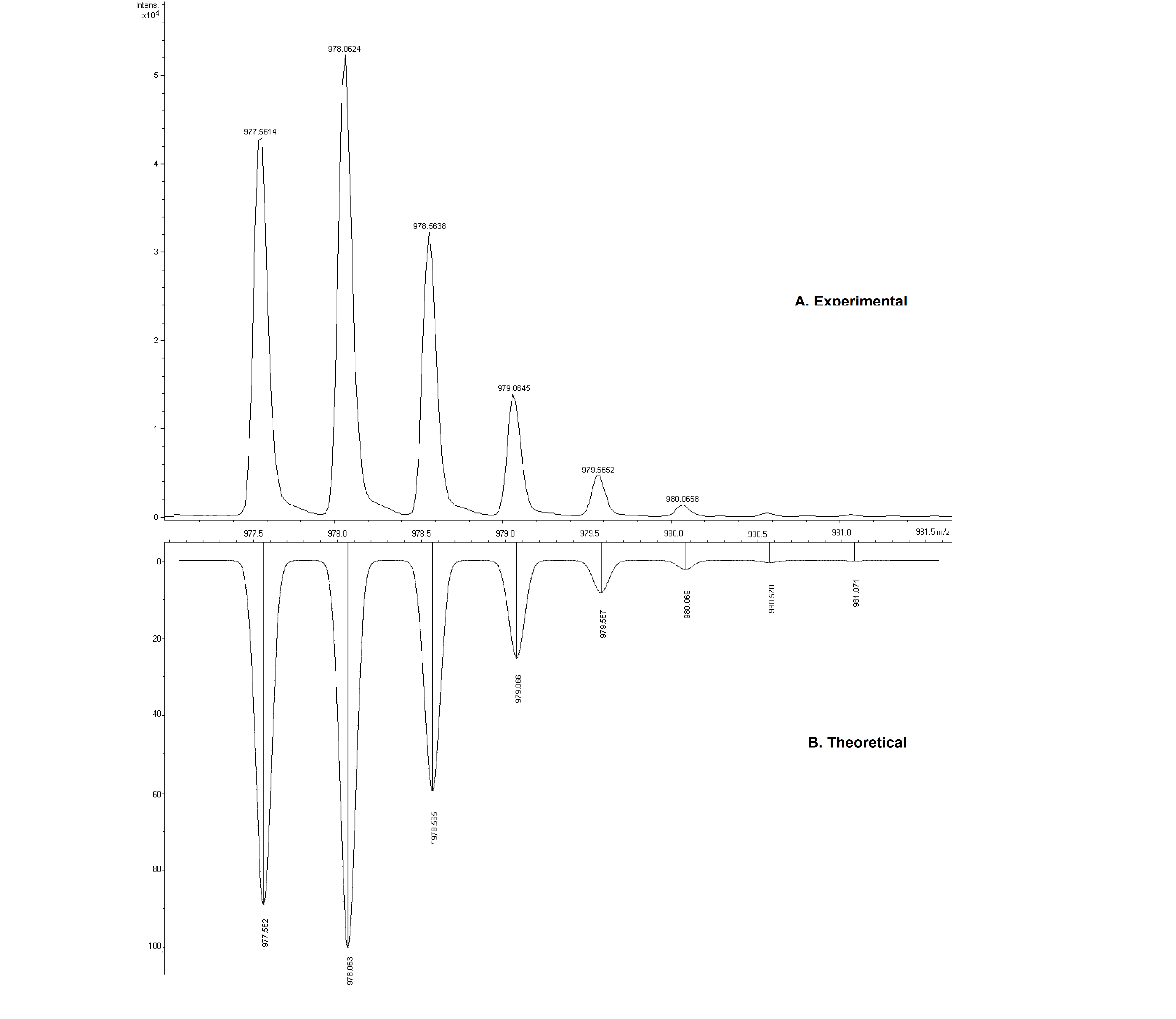

Supplement: Figure S2 — Theoretical and experimental accurate molecular mass values of Phenylseptins with internal calibration determined by direct infusion on an ESI MicrOTOF-Q II mass spectrometer operating with a standard ESI probe. Mass accuracy of <1 ppm RMS error; M+2H+ = 977.651. (TIF) [file pone.0059255.s002.tif]

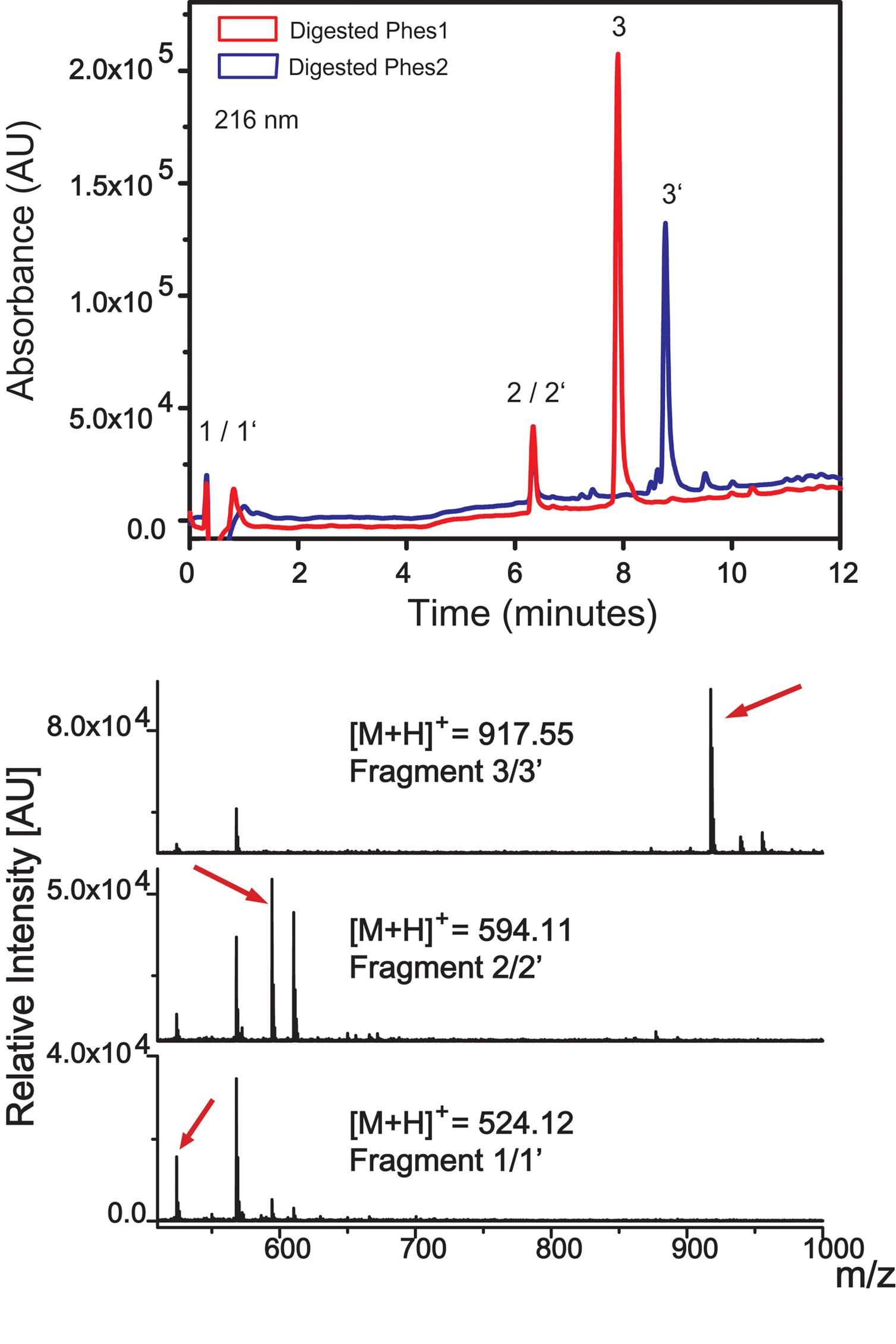

Supplement: Figure S3 — Enzymatic digestion of natural L-Phes and D-Phes with immobilized trypsin and UFLC analyses. (A) Analytical chromatographic profile of the digested peptides loaded onto an Ultra Fast Liquid Chromatography (UFLC-HPLC) using a Shimpack-XR-ODS column under a linear gradient of acetonitrile at a flow rate of 0.4 mL•min-1. L-Phes (red line) and D-Phes (blue line) generated 3 fragments each. Fragments 1/1′ and fragments 2/2′ were eluted at the same time, while fragments 3/3′ were eluted differently with a Δt = 0.9 min. (B) The molecular masses and purity of all fragments were determined by MALDI-TOF/MS (UltraFlex III, Bruker Daltonics, Germany) and the observed molecular mass was 524.1 Da for fragments 1/1′, 594.1 Da for fragments 2/2′ and 917.5 Da for fragments 3/3′. (TIF) [file pone.0059255.s003.tif]

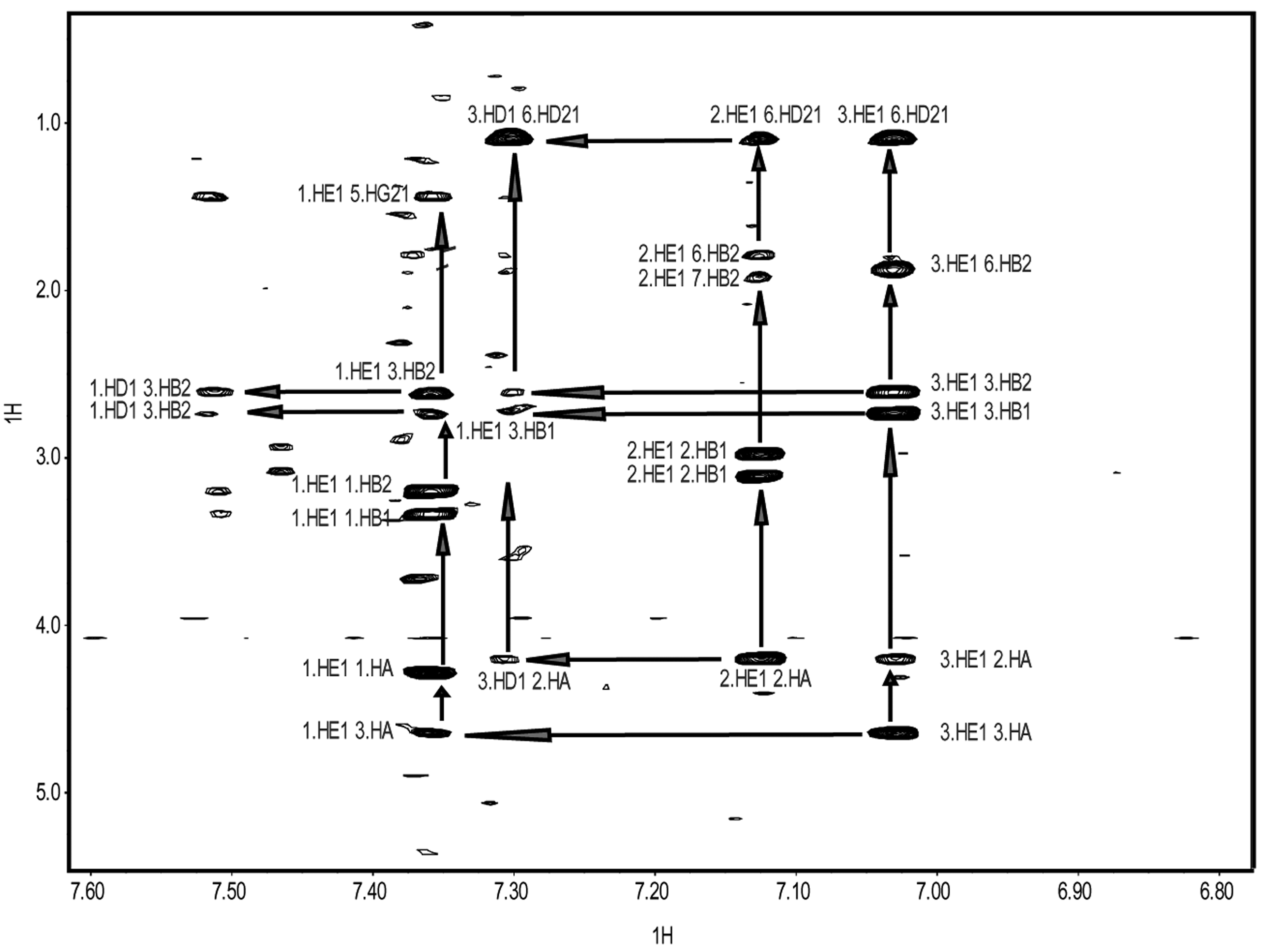

Supplement: Figure S5 — NOESY spectra acquired using mixing times of 160 ms from D-Phes showing Phe2 and Leu6 interaction. For this experiment, the acquisitions were carried out in 60% TFE/H2O (v/v). (TIF) [file pone.0059255.s005.tif]

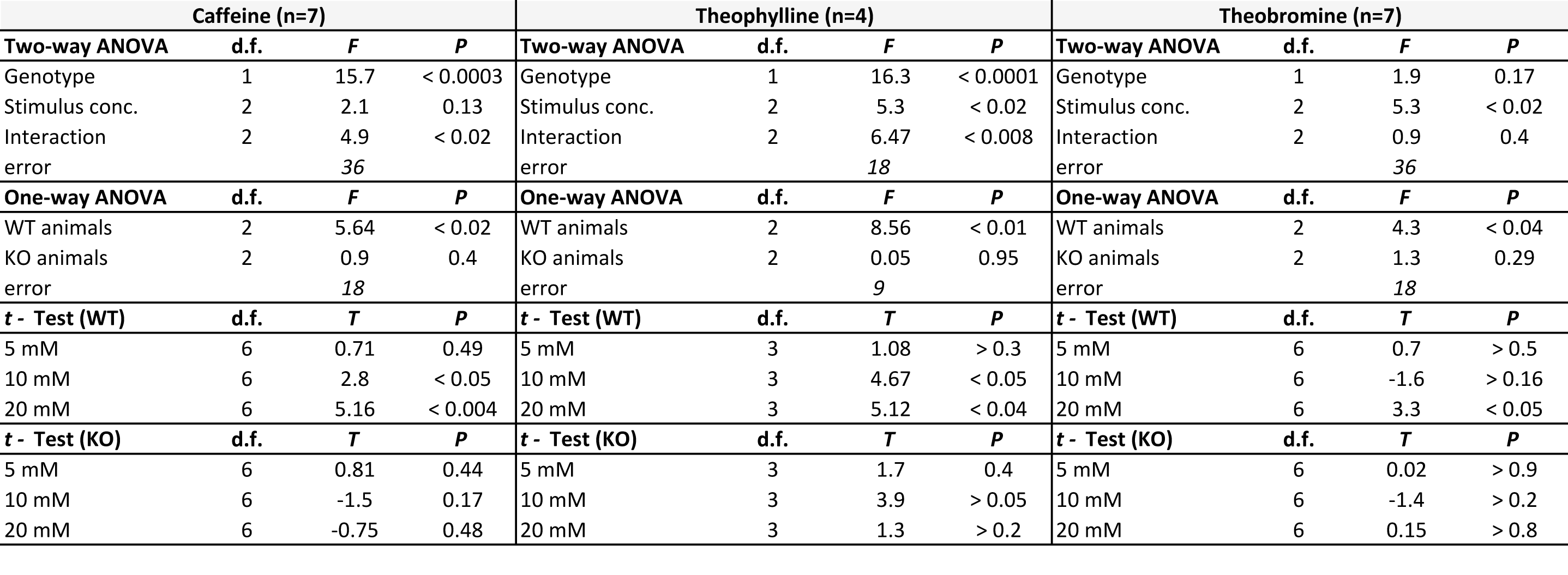

Supplement: Table S3 — (TIF) [file pone.0059255.s008.tif]
