# Supplementary material for: Conformational and Functional Effects Induced by D- and L-Amino Acid Epimerization on a Single Gene Encoded Peptide from the Skin Secretion of Hypsiboas punctatus
Source: PLoS One. 2013 Apr 2;8(4):e59255. doi: 10.1371/journal.pone.0059255 (PMC3614549; doi:10.1371/journal.pone.0059255)
Supplement: Table S1 — (DOCX) [file pone.0059255.s006.docx]

| **N-terminal*** | **Ion*** | **a _calc_** | **b _calc_** | **y _calc_** | **z _calc_** | **C-terminal *** | **Ion *** |
| --- | --- | --- | --- | --- | --- | --- | --- |
| **F** | **1** | **120.08** | **148.08** | **1954.12** | **1937.09** | **T** | **18** |
| **F** | **2** | **267.15** | **295.14** | **1807.05** | **1790.02** | **L** | **17** |
| **F** | **3** | **414.22** | **442.21** | **1659.98** | **1642.95** | **A** | **16** |
| **D** | **4** | **529.25** | **557.24** | **1512.91** | **1495.88** | **G** | **15** |
| **T** | **5** | **630.29** | **658.29** | **1397.88** | **1380.85** | **I** | **14** |
| **L** | **6** | **743.38** | **771.37** | **1296.84** | **1279.81** | **V** | **13** |
| **K** | **7** | **871.47** | **899.47** | **1183.75** | **1166.72** | **K** | **12** |
| **N** | **8** | **985.51** | **1013.51** | **1055.66** | **1038.63** | **G** | **11** |
| **L** | **9** | **1098.6** | **1126.59** | **941.61** | **924.58** | **A** | **10** |
| **A** | **10** | **1169.64** | **1197.63** | **828.53** | **811.5** | **L** | **9** |
| **G** | **11** | **1226.66** | **1254.65** | **757.49** | **740.46** | **N** | **8** |
| **K** | **12** | **1354.75** | **1382.75** | **700.47** | **683.44** | **K** | **7** |
| **V** | **13** | **1453.82** | **1481.82** | **572.38** | **555.35** | **L** | **6** |
| **I** | **14** | **1566.9** | **1594.9** | **473.31** | **456.28** | **T** | **5** |
| **G** | **15** | **1623.93** | **1651.92** | **360.22** | **343.19** | **D** | **4** |
| **A** | **16** | **1694.96** | **1722.96** | **303.2** | **286.2** | **F** | **3** |
| **L** | **17** | **1808.05** | **1836.05** | **232.17** | **215.14** | **F** | **2** |
| **T** | **18** | **1909.1** | **1937.09** | **119.08** | **102.05** | **F** | **1** |
| *** numbers and one-letter code amino acids refer to the peptide precursor lenght** | | | | | | | |
| **and to its respective residues in the sequence. Mass tolerance 0.1 Da** | | | | | | |  |
