# Supplementary material for: Conformational and Functional Effects Induced by D- and L-Amino Acid Epimerization on a Single Gene Encoded Peptide from the Skin Secretion of Hypsiboas punctatus
Source: PLoS One. 2013 Apr 2;8(4):e59255. doi: 10.1371/journal.pone.0059255 (PMC3614549; doi:10.1371/journal.pone.0059255)
Supplement: Table S2 — (DOCX) [file pone.0059255.s007.docx]

| Phes Sequence | Enzyme | Peptide Position | Fragment Sequence | [M+H]^+^  Calc* | [M+H]^+^  Exp | Hyd^b^ |
| --- | --- | --- | --- | --- | --- | --- |
| 1-7 | | | FFFDTLK | 917.46 | 917.55 | 2.51 |
| FFFDTLKNLAGKVIGALT-NH_2_ | Trypsin | 8-12 | NLAGK | 502.29 | 524.12^a^ | -2.16 |
| 13-18 | | | VIGALT-NH_2_ | 572.35 | 594.11^a^ | 2.53 |

* Calculated molecular mass values were determined by PeptideMass program available on Expasy (http://www.expasy.org/tools/peptide-mass.html)

a The molecular masses experimentally determined corresponding to [M+Na]^+^

b Calculated hydrophobicity values were determined according to CCS scale (<http://www.expasy.org/tools/peptide-mass.html>).
